# Environmental Sustainability in Dialysis Units: A Scoping and Integrative Review of Challenges and Innovations in Nephrology

**DOI:** 10.3390/healthcare14101284

**Published:** 2026-05-09

**Authors:** Abel Mata-Lima, Ana Rita Paquete, Herlander Mata-Lima

**Affiliations:** 1 (CTB-UPM) Center for Biomedical Technology (CTB), Universidad Politécnica de Madrid (UPM), 28223 Madrid, Spain; 2 Renal Division, Hospital Divino Espirito Santo (HDES), 9500-370 Ponta Delgada, Azores, Portugal; 3 (UFABC) Center for Engineering and Applied Social Sciences, Universidade Federal do ABC, São Paulo CEP-09280-560, Brazil

**Keywords:** sustainability, dialysis, carbon footprint, water consumption, energy use, waste generation, nephrology

## Abstract

**Background and Aims:** The global rise of chronic kidney disease (CKD) has led to a rapid expansion of dialysis services, which, although life-saving, are associated with substantial environmental costs. This study aims to analyze the environmental impacts of dialysis therapies and identify pathways toward more sustainable practices. **Methods:** This study conducts a comprehensive and integrative literature review on research in renal replacement therapy, adopting the PRISMA-ScR procedure to analyze papers published between 2005 and 2023. **Results:** The literature primarily focuses on four environmental dimensions: carbon emissions, water consumption, energy use, and waste generation. Dialysis therapies—particularly hemodialysis—are resource-intensive and contribute significantly to environmental degradation though repeated treatments, transportation requirements, and high consumption of materials and energy. **Conclusions:** Sustainable dialysis can be promoted through the adoption of green technologies, improved waste management, and policies focused on energy and resource efficiency. While current practices are environmentally demanding, feasible strategies exist to reduce their ecological footprint and align nephrology care with global sustainability goals.

## 1. Introduction

Chronic kidney disease (CKD) is on the rise globally, and millions of people rely on dialysis to survive end-stage renal disease (ESRD). Renal therapy processes have not experienced sustainable and substantial improvement for several decades, which is why many patients suffer a significant reduction in quality of life and a short life expectancy. In 2040, CKD is expected to be the 5th cause of death worldwide. Currently, 1 in 10 Europeans depend on renal replacement therapy (RRT) [1].

The current treatment options for ESRD include hemodialysis (HD), peritoneal dialysis (PD), and kidney transplantation. Although transplantation offers the best clinical outcomes, its availability is limited. As a result, dialysis remains the most widely used life-sustaining treatment [2].

However, dialysis therapies are highly resource-intensive [1,2,3]. They require large volumes of water, significant energy consumption, extensive use of single-use medical materials and generate substantial waste and greenhouse gas emissions. Consequently, dialysis contributes to environmental degradation and, indirectly, to adverse health outcomes [3,4].

In this context, it is essential to evaluate and mitigate the environmental aspects (EA) of dialysis therapies, as defined by ISO 14001 [5]. The environmental aspects refer to the elements of activities that interact with the environment or cause resulting changes, whether positive or negative.

Information and Communication technology (ICT) intervention can provide a chance to lead the charge in sustainable dialysis. A holistic approach is crucial to promote sustainability efforts to mitigate impact. Currently, there are several green practices to minimize the environmental aspects of the dialysis processes, but their implementation remains a concern because they require strong stakeholder engagement (e.g., continual education of staff is essential) and strong commitment from senior leadership [3,6].

This study aims to synthesize current evidence on the environmental footprint of dialysis and identify strategies to improve sustainability in nephrology care.

### Overview of Dialysis

The prevalence of patient dependance on dialysis is rising due to the global burden of CKD and an aging population. CKD affects about 850 million people worldwide and access to the therapy remains limited because of the high associated costs [6].

Hemodialysis (HD) dominates globally, making up 90% of dialysis treatments. However, HD is the least accessible and the most resource-intensive. Peritoneal dialysis (PD) usage varies widely; in countries such as Canada or New Zealand it reaches up to 30% of dialysis treatments and about 10% of dialysis patients in the United States are treated with it [7].

Due to the peculiarity of dialysis in the number of patients, its resource consumption (energy and water), the carbon emissions due to traveling and the huge amounts of waste generated, the environmental aspects of the dialysis process must be considered.

Nephrology care faces a big challenge due to climate-related strategies and, as highlighted by Yau et al., carbon emission generated by dialysis units goes beyond the process itself and is also due to patient and staff transportation [8,9,10,11].

As a contribution to the body of knowledge this research establishes a picture of resource consumption (water and energy), waste generation, and carbon emissions of dialysis in light of the following sustainable development goals (SDGs): industry, innovation and infrastructure (SGD 9), responsible consumption and production (SDG 12), and climate action (SDG 13) [12,13,14,15].

These SDGs were selected because they directly relate to healthcare sustainability:

SDG 9 (industry, innovation and infrastructure) addresses technological innovation in dialysis systems; SDG 12 (responsible consumption and production) relates to resource use and medical waste; SDG 13 (climate action) addresses greenhouse gas emissions from healthcare activities.

The growing interest in sustainable healthcare is also reflected in global policy frameworks such as the United Nations Sustainable Development Goals (SDGs). SDG 9 (industry, innovation and infrastructure), SDG 12 (responsible consumption and production), and SDG 13 (climate action) are highly relevant to the environmental sustainability of healthcare systems. These goals emphasize the importance of resource efficiency, technological innovation, and climate mitigation strategies in healthcare delivery.

The purpose of this study is to fill the literature gap by analyzing and synthesizing current research comparing the environmental impact of dialysis practices, regarding the environmental aspects mentioned above (e.g., energy consumption, water usage, carbon emissions, and waste generation). This work provides a roadmap for dialysis providers, policymakers, and other stakeholders to implement eco-friendly practices in renal care [16].

Several reviews have examined the environmental impact of dialysis therapies; however, these studies often focus on single dimensions such as carbon emissions or water usage or are limited to specific geographic contexts. In addition, recent technological developments and sustainability initiatives in nephrology have not been comprehensively synthesized.

This study adopts a scoping review methodology following the PRISMA-ScR guidelines, while incorporating integrative analytical elements. Beyond mapping the scope and characteristics of the literature, the review also synthesizes comparative evidence on environmental impacts across dialysis modalities and identifies emerging technological and policy responses. This dual approach enables both descriptive and interpretive insights into sustainability in nephrology.

This review addresses the following research questions:

What is the scope, distribution, and thematic focus of the literature on environmental sustainability in dialysis?
Which environmental dimensions (carbon emissions, water use, energy consumption, waste generation) are most frequently studied, and where are the main gaps?
How do different dialysis modalities (hemodialysis, peritoneal dialysis, home dialysis) compare in terms of environmental impact?
What technological innovations, policies, and practices have been proposed to reduce the environmental footprint of dialysis?
What are the key limitations of the current evidence and priorities for future research?

## 2. Methodology

For this research, the first step was to search for research that covered the environmental aspects of RRT. The search was conducted in PubMed, Scopus, and Web of Science to identify studies addressing environmental sustainability in dialysis therapies. The search combined terms related to dialysis and environmental impact, including: “dialysis”, “hemodialysis”, “peritoneal dialysis”, “carbon footprint”, “climate change”, “water consumption”, and “healthcare sustainability”.

The review followed (PRISMA-ScR) guidelines and included studies published between 2005 and May 2023. The review was registered and the project DOI is 10.17605/OSF.IO/J7UGV.

We started from the year 2005 to consider studies affected by more recent technologies, considering multiple electronic databases, and did not limit the search by the language, aiming to ensure comprehensive coverage of relevant studies.

Four significant environmental aspects were considered: carbon emissions, water usage, energy consumption and waste generation. Two main modalities of dialysis treatment were included (HD and PD). Studies that assessed several environmental aspects stemming from the use of resources, energy consumption, and waste disposal were selected.

The search started using the expression “Effects dialysis therapies on climate change”. To refine the search and focus on the studies concerning the identified environmental impact factors, we decided to continue the search using the following queries: (“Green Dialysis”) OR (“Sustainable practices in dialysis”) OR (“Environmental impact factors” AND “Dialysis”) OR(“Dialysis” AND “Climate Change”) OR (“Dialysis” AND “Climate Change”) OR (“Dialysis” AND” Carbon Footprint”) OR (“Dialysis” AND “Water Consumption”) OR (“Dialysis” AND “Water usage”) OR (“Dialysis” AND “Waste Generation”).

Data extraction was independently performed by the authors to evaluate the study quality and extract characteristics, study title, author and year, geographic scope, environmental aspect, key findings, limitations and future research. Reference lists from relevant review articles were also searched. Studies were first screened according to title and abstract, and the full text of any studies considered relevant according to the selection criteria were assessed for eligibility by the authors.

Then, studies on the environmental aspects of dialysis were identified and analyzed. Data extraction and analysis were conducted systematically to ensure the reliability and validity of the findings.

### 2.1. Study Selection

The study selection, assessment of eligibility criteria, and data extraction were performed independently by two authors (AM-L and ARP). To mitigate selection bias, rigorous inclusion and exclusion criteria were established previously. In addition, precise search strategies were formulated to better identify all relevant papers regarding the research scope.

Both authors screened titles, abstracts, and full texts to determine the study eligibility, thus reducing the risk of selection bias.

### 2.2. Data Extraction

Prior to data extraction, the authors defined a standardized data extraction strategy to ensure consistency and completeness in data collection. The strategy was designed to collect the key findings and environmental aspects of each study, geographic scope, limitations and future research directions, and other relevant details for this study. The extracted data were cross-verified to ensure consistency and accuracy.

### 2.3. Exclusion Criteria

Studies were excluded based on the following criteria:

- Duplicated studies;
- Non-peer-reviewed or preprint publications such as conference abstracts, opinion pieces, or letters for editors;
- Incomplete reporting of outcomes or studies with insufficient data for extraction
- Studies that do not report outcomes directly relevant for the environmental issues under review;
- Studies with a lack of focus on the identified environmental issues;
- Animal studies.

### 2.4. Screening Process

**Figure 1:**
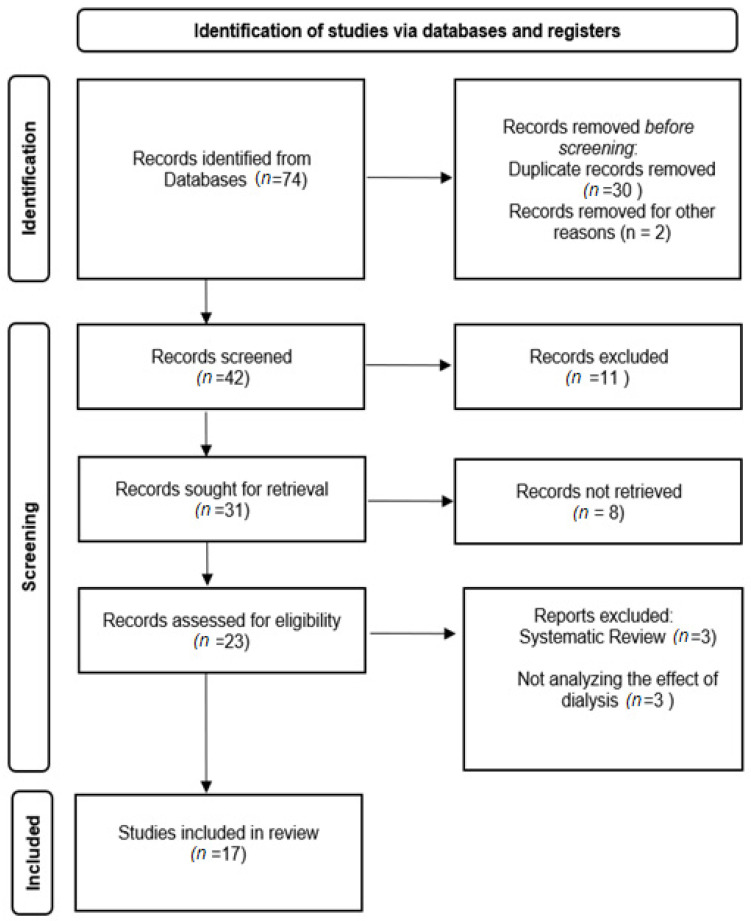
Flow chart of search and study selection process.

After the initial search, all identified records were exported and analyzed for duplicate removal. The screening process was conducted as described in Figure 1.

The search strategy yielded 74 records (see Figure 1). After removing duplicates, 62 records were screened. A total of 39 studies were excluded based on title/abstract screening. In total, 23 full-text articles were assessed for eligibility, of which 6 were excluded, resulting in 17 studies included in the final analysis. Additionally, 8 records could not be retrieved due to unavailable full texts.

Besides the 17 articles considered for the scope review, a few other documents (10) were considered to explain the environmental terms and ESRD treatments, as well as to subsidize discussions.

## 3. Results

The 17 included studies show substantial heterogeneity in their geographic scope, methodological approaches, and environmental indicators assessed (Appendix A Table A1).

Most studies were conducted in high-income countries, particularly Australia (*n* = 4), followed by the United States, Germany, Poland, and France (*n* = 2 each), with limited representation from low- and middle-income countries.

In terms of environmental dimensions, carbon emissions were the most frequently assessed (*n* = 15), followed by water usage (*n* = 14), energy consumption (*n* = 13), and waste generation (*n* = 12). However, few studies examined all four dimensions simultaneously, and methodological approaches varied widely, including life-cycle assessments, facility-based measurements, and modeling studies.

This variability limits direct comparison across studies and highlights the absence of standardized metrics in this field.

Methodologically, studies varied widely, including observational assessments, modeling approaches, and facility-based evaluations. This variability limits direct comparability across studies and highlights the absence of standardized frameworks for assessing environmental sustainability in dialysis.

Most studies were published after 2020, with a peak in 2022 (40%).

### 3.1. Distribution of Environmental Dimensions

The field of nephrology presents a significant environmental challenge, requiring close collaboration between healthcare providers and policymakers to achieve green targets [17].

The largest contributors to total emissions are patient and staff transportation (28.3%), electricity use (27.4%), and natural gas consumption (15.2%) [17]. The environmental costs of transportation, especially for shipping ready-to-use dialysis concentrates, are high, and require re-evaluation to reduce the carbon impact [3,17,18,19]. While dialysis is a life-saving treatment, it has a significant negative impact on environmental success, potentially affecting billions’ health and well-being [20,21,22].

Carbon footprint analysis dominates the literature, reflecting growing concern about healthcare-related greenhouse gas emissions.

Water usage and energy consumption are also widely studied, particularly in relation to hemodialysis infrastructure and processes. Waste generation, although frequently acknowledged, is less consistently quantified.

### 3.2. Methodological Gaps and Limitations in the Literature

The reviewed literature reveals several important methodological limitations.

First, there is a lack of standardized metrics and system boundaries across studies, which complicates comparisons of environmental performance between dialysis modalities and healthcare settings. Second, most studies focus on operational phases (e.g., in-center dialysis processes), while upstream and downstream impacts—such as manufacturing, transportation, and waste disposal—are often underrepresented.

Additionally, few studies employ full life-cycle assessment (LCA) methodologies, limiting the ability to capture the total environmental footprint of dialysis systems. Geographic concentration in high-income countries further restricts the generalizability of findings to resource-constrained settings.

### 3.3. Comparative Environmental Impacts of Dialysis Modalities

The reviewed evidence consistently indicates that hemodialysis (HD) is the most resource-intensive dialysis modality.

Hemodialysis showed the highest environmental burden. Water use ranged from 300 to 500 L per session. Carbon emissions ranged from 15 to 35 kg CO_2_e per session, depending on methodology and infrastructure, Energy use was consistently high due to machine operation and water treatment systems.

Waste generation was substantial, largely due to single-use consumables. Transportation of patients and supplies was a major contributor to emissions.

The main dialysis modalities are HD and PD, and both contribute to environmental problems. PD is a home-based renal replacement therapy (RRT), whereas HD is still currently mostly performed in-center, but efforts to increase the number of patients being treated with home hemodialysis (HHD) are being strengthened. Although HHD promises improvement in patient outcomes and reductions in harmful effects on the environment, it will continue to be an underutilized RRT [8].

Kidney transplantation is the best and cheapest therapy for ESRD, followed by PD and HD. However, kidney transplantation is the least performed because of the few organs available for transplant [6,7].

PD uses plastic-based dialysate solutions and produces a lot of waste. On the other hand, HD treatment is a notable energy consumer, and involves high ultrapure water usage and waste generation, with high environmental impact and resource consumption [6,8,9].

The findings also reveal that high ambient temperature increases the risk of renal problems. On the other hand, the RRT available currently has environmental aspects that contribute negatively to climate change because of the large amount of resources demanded [9].

The values presented in Table 1 are approximate ranges derived from multiple studies included in this review and are intended to provide an indicative comparison rather than statistically pooled estimates [3,8,10,13,17,18,19,23,24,25,26,27].

### 3.4. Key Drivers of Environmental Impact

Across the reviewed studies, several key drivers of environmental impact were consistently identified.

Transportation of patients and supplies represents a major contributor to carbon emissions, accounting for a substantial proportion of the total footprint in many settings. Energy consumption, particularly electricity and natural gas used in dialysis facilities, is another dominant factor.

Water use, especially in HD, is driven by the need for high-purity dialysate and results in significant volumes of rejected water. In addition, the widespread reliance on single-use consumables contributes to both waste generation and upstream environmental impacts related to manufacturing and disposal.

### 3.5. Emerging Strategies and Methodological Innovations

The literature identifies multiple strategies to reduce the environmental footprint of dialysis.

These include the adoption of energy-efficient technologies, implementation of water recycling systems, reduction of single-use materials, and improved waste segregation and recycling practices. Technological innovations in dialysis machines and water treatment systems have demonstrated the potential to reduce water and energy consumption by up to 30% in some settings [13,23,28].

Home-based therapies and tele-nephrology have also been proposed as approaches to reduce transportation-related emissions. However, these strategies require careful evaluation using life-cycle perspectives to account for potential trade-offs.

### 3.6. Impact of Transportation and Home Dialysis Alternatives

Home dialysis can significantly reduce transportation-related emissions. Peritoneal dialysis also has a different resource consumption profile and is more adaptable to lower-environmental-impact systems, such as in the reduction of the use of running water and solar energy [29,30].

Home-based dialysis therapies have been proposed as a strategy to reduce environmental impacts, particularly by decreasing patient transportation to dialysis centers.

However, home dialysis may introduce additional environmental considerations, including increased household electricity use, water consumption, and packaging waste associated with home delivery of dialysis supplies.

Future studies should evaluate the full life-cycle environmental aspects of home dialysis compared with in-center dialysis, considering both healthcare system and household resource consumption.

Carbon emissions associated with dialysis treatments vary widely depending on the energy sources, healthcare infrastructure, and methodological approaches used to calculate emissions.

**Figure 2:**
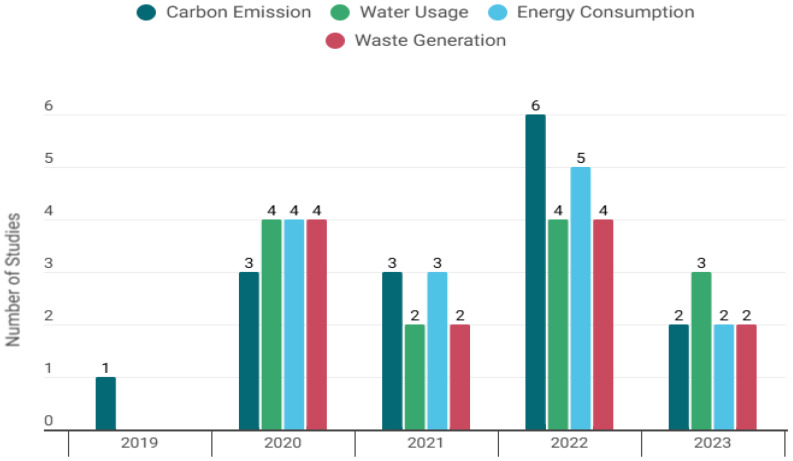
Annual distribution of the environmental aspects considered in the studies.

Figure 2, shown below, presents the annual distribution of the environmental aspects Figure 2, shown below, presents the annual distribution of the environmental aspects considered in the studies, by year.

Few studies apply full life-cycle assessment approaches, with most focusing on operational phases. Additionally, inconsistencies in system boundaries, units of measurement, and reporting standards limit comparability.

## 4. Discussion

This review highlights the significant environmental aspect of dialysis, particularly HD. The variability of studies suggests a lack of standardized assessment methods.

Strategies to improve sustainability include:

- Energy-efficient technologies;
- Water recycling systems;
- Reduction of single-use materials;
- Optimization of logistics.

Home-based therapies may reduce transplantation emissions but introduce other environmental trade-offs. Life-cycle assessment approaches are needed.

### 4.1. Summary of Main Findings

This review highlights the substantial environmental impact of dialysis therapies, particularly hemodialysis, which consistently emerges as the most resource-intensive modality across all the environmental dimensions examined.

The findings indicate that dialysis care is associated with high levels of water consumption, energy use, carbon emissions, and waste generation. At the same time, the literature remains fragmented, with significant variability in methodologies and limited use of standardized assessment frameworks.

Transportation, energy consumption, and single-use consumables were identified as the primary drivers of environmental impact, underscoring the systemic nature of sustainability challenges in nephrology [8,10,17].

### 4.2. Contribution to the Existing Literature

This study extends previous reviews by providing an integrated analysis of multiple environmental dimensions and comparing dialysis modalities within a single framework.

While earlier studies have often focused on individual aspects such as carbon emissions or water use, this review highlights the interconnected nature of environmental impacts in dialysis systems. It also emphasizes the importance of considering both direct and indirect environmental aspects, including supply chains and infrastructure.

By combining scoping and integrative approaches, this review offers a more comprehensive understanding of sustainability challenges and opportunities in dialysis care [3,13,18].

### 4.3. Policy and System-Level Implications

The findings have important implications for healthcare policy and system design.

Improving the environmental sustainability of dialysis requires coordinated action across multiple levels, including infrastructure development, procurement practices, and care delivery models. Policies promoting energy efficiency, renewable energy use, and sustainable procurement of medical supplies can significantly reduce environmental impacts.

In addition, encouraging the adoption of home-based therapies where clinically appropriate may help reduce transportation-related emissions. However, these strategies should be supported by appropriate infrastructure and evaluated within a life-cycle framework.

### 4.4. Technological Innovations and Opportunities

Technological innovation plays a critical role in advancing sustainable dialysis. Based on Mata-Lima et al., direct and indirect environmental aspects can be defined respectively as the ones that can be influenced and controlled by the dialysis unit and influenced but not controlled by the dialysis unit, because they come from supplier activities, products or services [16,17,18].

Recent developments in dialysis machines and water treatment systems have demonstrated significant potential for reducing resource consumption. Innovations such as dialysate regeneration, water recycling, and solar-powered peritoneal dialysis systems offer promising pathways toward more sustainable care.

However, the adoption of these technologies remains uneven, and further research is needed to evaluate their long-term environmental and economic performance.

### 4.5. Research Gaps and Future Directions

This review identifies several key gaps in the current evidence base.

First, there is a need for standardized metrics and reporting frameworks to enable meaningful comparisons across studies and settings. Second, more comprehensive life-cycle assessments are required to capture the full environmental impact of dialysis systems [3,13,18,19,20].

Third, there is a significant lack of data from low-and middle-income countries, where resource constraints and infrastructure differences may lead to distinct environmental challenges and opportunities.

Future research should also incorporate economic evaluations of sustainability interventions to support evidence-based decision-making in healthcare systems [21].

### 4.6. Limitations

This review has several limitations. First, the number of studies included is relatively small *(n* = 17), reflecting the emerging nature of this field of research and limiting the generalizability of findings. Second, substantial heterogeneity exists across studies in terms of methodologies and system boundaries, and environmental heterogeneity exists across studies in terms of their methodologies, system boundaries, and environmental indicators, which restricts direct quantitative comparison and precludes meta-analysis.

Third, many studies rely on partial assessments of environmental impact (e.g., focusing only on operational phases), with limited use of full life-cycle assessment approaches. As a result, upstream and downstream impacts—such as manufacturing, transportation, and end-of-life disposal—may be underrepresented. Fourth, most studies originate from high-income countries, which may limit the applicability of findings to low-and middle-income settings where infrastructure, energy mixes, and resource constraints differ significantly [22,28].

Finally, reported ranges for environmental indicators (e.g., carbon emissions, water use) are derived from heterogeneous sources and should be interpreted as indicative rather than definitive estimates. Future research using standardized frameworks and transparent reporting is needed to strengthen the evidence base.

### 4.7. Carbon Emissions: Urgency of a Green Dialysis Approach

Dialysis generates a considerable carbon footprint, with emissions mainly derived from the use of electricity to power dialysis machines, water treatment (ultrapure water and wastewater), maintaining appropriate temperature and humidity levels in dialysis facilities, lighting the rooms, and powering the other facility equipment [29,31]. Transportation also contributes to the carbon footprint, because of the transportation of medical supplies and patients’ need to travels to and from treatment centers several times a week, often using gas cars or ambulances [13,17,31,32,33]. Other contributions come from single-use medical supplies and the corresponding packaging which used to be non-recyclable, from biohazardous waste, and pharmaceutical product manufacturing and distribution [32].

Home dialysis (HD) can significantly reduce emissions concerning transportation. PD, in addition to being a home therapy, has a different profile of resource consumption and is more easily adaptable to systems with minor environmental impact, by reducing water usage and energy consumption. Currently, most dialysis consumables are single-use, without the option for recycling, reuse or biodegradation. The consequence is the generation of large amounts of medical waste. Studies demonstrate that staff training can reduce medical waste from about 2 kg to 1 kg per treatment session. This reduction has the potential to save about 100,000 t of carbon dioxide. Reducing emissions represents a significant economic challenge for the healthcare system [13,27].

It is crucial that patients, healthcare providers, researchers and policymakers work together to promote green initiatives. This includes improving infrastructure design and waste management practices, optimizing energy use, and prioritizing sustainability in treatment planning and facility design [33].

### 4.8. Need for Climate Change Preparedness

In the face of extreme weather events and rising temperatures, healthcare professionals must educate patients about the related risks and prepare them to adapt to these situations.

Due to direct and indirect environmental aspects, moving towards greener dialysis requires immediate action and collaboration among all sectors involved. Another relevant issue of major concern is the need for future research to explore technological improvements and resource management strategies, considering the limitations observed in current studies.

Based on the included studies, the recommendations for future studies include saving water and energy, increasing equipment efficiency, and reducing the carbon footprint of dialysis [24,33].

Since the main sources of emissions are electricity, gas and transportation for patients, consumables, and staff, training healthcare workers can reduce the significance of environmental aspects of dialysis [34].

Recent advancements in peritoneal dialysis (PD), including systems that produce dialysate from tap water and operate on solar energy, have helped lower its carbon footprint. Both peritoneal dialysis and kidney transplantation may present environmental benefits compared to hemodialysis [34,35,36].

Primary care, in collaboration with nephrologists, should strengthen efforts to cut treatment costs through energy and material efficiency. This strategy might allow for more resources to be directed toward improving patient care and outcomes [21,37].

Key areas for improvement of EA include organizational culture, infrastructure, facility design, energy consumption, waste management, and treatment frequency [38,39].

Some studies indicate that renal replacement therapies (RRT) are among the most resource-intensive medical technologies [40,41]. Hence, to address the shortage of sustainable initiatives in dialysis units, collaboration among researchers, patients, healthcare providers, and policymakers will be necessary.

HD is particularly demanding in terms of water usage, though some practices have successfully reduced water consumption by half. Energy use, however, varies depending on the treatment setting (home versus in-center) and the equipment utilized. Dialysis sessions require substantial amounts of water and electricity, and produce considerable medical waste, making effective management of chemical use and disposal in the dialysis process an issue of major concern [41,42].

### 4.9. Future Directions and Research Needs

It is also important to take measures to manage the use and disposal of chemicals in the dialysis process and develop a shared global guideline to minimize environmental impacts, and delay or even avoid dialysis needs.

### 4.10. Resource Optimization

Hemodialysis costs and practices must be evaluated, including patient transport and waste management, to reduce costs and improve sustainability [18,23].

To work on the limitations, in future research it is necessary to focus on CKD prevention. Each dialysis center must implement measures to follow dialysis indicators based on defined standards and develop plans to define multidisciplinary annual follow-up.

The development of new technologies to create innovative methods for dialysate regeneration and waste management, as well as exploration of the connections between climate change and adverse effects on patients with chronic kidney disease is crucial [11,30,43].

A notable limitation is the geographical concentration of the studies in high-income countries, particularly in Europe, Australia, and the United States. Only three studies were conducted in low-and middle-income countries (LMICs). Since dialysis infrastructure, resource availability, and energy systems vary significantly across regions, the generalizability of the results to LMIC contexts may be limited [44,45,46,47].

### 4.11. Home Dialysis Trade-Offs

While home dialysis helps reduce transportation-related emissions, it can also lead to an increase in household electricity consumption and packaging waste from supplies delivered to the home, underscoring the need for life-cycle assessments [34,48].

### 4.12. Economic Implications of Green/Sustainable Dialysis

The implementation of sustainability strategies in dialysis units may also have economic implications. Measures such as water reuse systems, improved waste segregation, and energy-efficient equipment may require initial investment but could generate long-term cost savings through reduced resource consumption.

Although many sustainability interventions have been proposed for dialysis units, limited evidence exists regarding their economic implications.

Strategies such as water recycling systems, improved waste segregation, and energy-efficient dialysis equipment may require initial investments but could potentially generate long-term cost savings through reduced resource consumption and waste management costs [46,47,49].

Further research evaluating the cost-effectiveness of environmental sustainability interventions in dialysis care would support healthcare decision-making and policy development.

## 5. Conclusions and Recommendations

Dialysis therapies are essential for sustaining patients with end-stage renal disease but are associated with significant environmental aspects, including high water consumption, energy demand, waste generation, and greenhouse gas emissions. The synthesized evidence indicates that meaningful reductions are achievable through resource-efficient technologies, optimized logistics, improved waste management, and broader adoption of home-based modalities where appropriate.

To enable robust comparisons and guide policy, future studies should adopt standardized metrics and life-cycle assessment (LCA) approaches, incorporate economic evaluations, and expand evidence from low-and middle-income countries. Embedding sustainability into nephrology practice is essential to align life-saving care with environmental responsibility.

Addressing these challenges requires coordinated action across clinical practice, healthcare policy, and industry innovation. Sustainable strategies—such as improving resource efficiency, adopting renewable energy, promoting home-based therapies, and developing recyclable materials—can substantially reduce the environmental footprint of dialysis care.

Future research should expand the evidence base by incorporating life-cycle assessment methodologies, economic evaluations, and studies conducted in low-and middle-income countries. Integrating sustainability principles into nephrology care will be essential to ensure that life-saving dialysis treatments can be delivered in a manner consistent with global climate and environmental goals.

## Figures and Tables

**Table 1:** Significance of environmental aspect of RRT.

Environmental Indicator/Aspects	HD	PD	Key Drivers
Carbon emissions	15–35 kg CO_2_e/session *	18–40 kg CO_2_e/week equivalent *	Consumables and transport
Water usage	300–500 L/session *	Minimal direct water use	RO reject water
Energy consumption	High (machines + RO)	Moderate	Home electricity
Waste generation	High (single-use plastics)	Moderate-high	Packaging

RO: Reverse osmosis; CO_2_e: Carbon dioxide equivalent; * Indicative ranges from multiple studies.

## Data Availability

No new data were created or analyzed in this study.

## Appendix A

The list of included articles is available below (Table A1).

**Table A1:** Characteristics of studies included in this review.

	Study Title	First Author and Year	Geographic Scope	Environmental Indicators				Key Findings/Results	Limitations and Future Research Directions
				Carbon Footprint/Transportations	Water Usage	Waste Generation	Energy Consumption		
01	“Water Implications In Dialysis therapy, Threats and opportunities to reduce water consumption; a call for the planet”	Ben Hmida M. et al. (2023) [4]	Tunisia		x	x		Very high water usage. Develop more efficient dialysis machines and new hardware.	Further studies are required to assess water usage, energy needs, equipment performance and carbon footprint. Share global guidelines to minimize the environmental impact. Environmental certification should be required for dialysis units.
02	“Advanced hemodialysis equipment for more eco-friendly dialysis”	Gauly A. et al. (2022) [3]	Germany	x	x	x	x	High water and energy consumption during the production process. Manufacturers must produce dialysis systems and consumables economically and ecologically. Dialysis systems and resource processes need improvements. Waste management needs improvement. Online treatment can reduce environmental effects.	Promote environmental management systems. Implement education and tracking programs for all stakeholders in dialysis care to monitor needs and achievements. Choose the right place and right modality for treatment.
03	“Informed decision-making in delivery of dialysis: combining clinical outcomes with sustainability”	Apel C. et al. (2021) [18]	Argentina/Hong Kong/Germany	x	x	x		Water protection. Atmosphere protection. Resource conservation.	Healthcare providers need to consider: Cost of HD treatment; Cost of transport; Cost of waste disposal.
04	“Sustainable kidney care delivery and climate change–a call to action”	Yeo S. C. et al. (2022) [28]	Singapore	x	x	x	x	All dialysis providers must recognize the relevance and urgency of the impact of hemodialysis on climate change. Immediate and clear contributions are needed to achieve more sustainable hemodialysis provision and kidney care delivery.	Assessment of more sustainable hemodialysis provision and kidney care delivery.
05	“Green Dialysis: Let Us Talk about Dialysis Fluid”	Zawierucha J. et al. (2023) [23]	Poland	x	x		x	The environmental costs of transportation are significant. The transportation of ready-to-use liquid concentrate generates a higher carbon footprint in comparison with other options. The increasing cost of fuel, manpower, and water forces a change in behavior and creates consequences for routine procedures.	It is necessary to rethink some common rules, e.g., regarding dialysate flow, and to develop modern systems to help dialysis professionals be sensitive to environmental issues.
06	“Sources of Variation in the Carbon Footprint of Hemodialysis Treatment”	Sehgal A. R. et al. (2022) [17]	USA	x			x	The three largest contributors to total emissions were patient and staff transportation (28.3%), electricity (27.4%), and natural gas (15.2%). The contributors with the largest variation in emissions per treatment were transportation, natural gas, and water (coefficients of variation, 62.5%, 42.4%, and 37.7%, respectively). Annual emissions per HD facility are the same as the annual energy usage of 93 homes. Emissions per treatment correspond to driving an automobile for 238 Km.	Find a way to identify measures to reduce the environmental effect of hemodialysis treatment.
07	“A call-to-action for sustainability in dialysis in Brazil”	Moura-Neto J.A. et al. (2019) [22]	Brazil	x				Dialysis is an established treatment that, while saving millions of lives, paradoxically causes great negative impact to the environment that may affect the health and well-being of billions of people.	To spread the word of the necessity of sustainability in dialysis to create a collective consciousness of green dialysis. HCPs need to develop an environmentally positive attitude towards a sustainable future.
08	“Haemodialysis therapy and sustainable growth: a corporate experience in France”	Bendine G. et al. (2020) [13]	France	x	x	x	x	From improvements in dialysis technology (dialysis machines and water treatment systems) and from updating and remodeling of dialysis unit equipment and buildings we can achieve a 52% reduction in power consumption and a 29.6% reduction in water consumption. Staff training reduces care-related waste from 1.8 kg to 1.1 kg. These savings have been estimated as equivalent to 102,440 t of carbon dioxide.	Additional measures related to companies’ responsibilities and individual behaviors must be implemented to allow sustainable growth, because the demand for dialysis therapy is growing quickly around the world. The nephrology community must be sensitized to this challenge and must be proactive to anticipate future regulations.
09	“Carbon Footprint of Hemodialysis unit in Morocco”	Mtioui N. et al. (2021) [50]	Morrocco	X				Decarburization comes at a high economic cost. We should reduce the cost of treatment through the efficient use of energy and consumables, which in turn will allow redirection of savings to clinical care and better outcomes for patients.	For all future carbon emissions research in dialysis, the involvement of environmental scientists is critical to ensure robust data collection and analysis at a fine level of detail.
10	“A Survey of Environmental Sustainability Practices in Dialysis Facilities in Australia and New Zealand”	Talbot B. et al. (2022) [43]	Australia and New Zealand	x	x	x	X	Environmental sustainability is not currently prioritized in clinical practice, building design and infrastructure, or management systems The following domains of opportunity for improvement in environmental sustainability strategy were identified: culture, building design, infrastructure, energy use, and waste management.	
11	“Assessing the Carbon Footprint of Hemodialysis: A First Step Toward Environmentally Sustainable Kidney Care”	Barraclough, K.A et al. (2022) [10]	Australia	x	x	x	x	The carbon footprint of HD is particularly high due to the high use of energy, water, consumables, and repeated nature of dialysis treatment. The largest contributors were electricity and gas usage and transport of both patients and staff. Home dialysis can mitigate emissions from transport. Peritoneal dialysis has very different resource consumption to other methods.	Further work must still be done to understand water usage in dialysis units. Water and energy sub-metering equipment should be installed to facilitate future study and unit management.
12	“Water use in dialysis: environmental considerations”	Agar, J.W.M et al. (2020) [14]	Australia	x	x	x	x	Performance in environmental sustainability should be considered by renal services when negotiating contracts with provider companies. Companies providing dialysis equipment should be required to provide carbon-equivalent estimates of the product(s) they make and sell.	Young researchers should be encouraged to enter and develop this understudied research field, and will do a better job with the “back-end detritus” that our healthcare sector leaves behind than those who have gone before them.
13	“Environmental Impact of Care for End-stage Kidney Disease on the Earth and Humans”	Nagai K. et al. (2021) [29]	Japan	X	X		X	Annual dialysis procedures for 418 patients could represent 1-year disability-adjusted losses.	Peritoneal dialysis and renal transplantation are expected to have fewer negative environmental effects than hemodialysis. A large field survey is needed to ensure the reliability of this study’s findings.
14	“Eco-dialysis: fashion or necessity”	Wieliczko M. et al. (2020) [30]	Poland and Romania	x	x	x	x	Renal replacement therapies are one of the most resource-consuming medical technologies. A large amount of water and electricity is needed to provide a dialysis session. Every HD session produces a significant amount of medical waste. HD treatment generates seriously high costs for the healthcare system and waste effects on the environment are undisputed.	Urgent research efforts are needed to study dialysate regeneration and waste disposal by new methods. Alongside nephrologists and their change of thinking, the industry should increase their efforts in the development of more “green” dialysis products.
15	“Climate and the Nephrologist: The Intersection of Climate Change, Kidney Disease, and Clinical”	Young S. E. et al. (2023) [31]	USA	x	x	x	x	Environmental effects occur in three areas: water use, energy consumption, and waste management. The “carbon footprint” of dialysis can also be estimated. HD is an extremely water-hungry treatment. There have been successful efforts to reduce water usage in dialysis. Energy use is dependent on both the method (home versus in-center) and machine. Recent innovations in PD to reduce its carbon footprint include a PD system that produces pure water for dialysate and runs on solar power and methods to generate dialysate from tap water. Nephrologists must redouble efforts to prevent and slow progression of CKD, considering its effect on the climate.	There is a scarcity of studies evaluating the environmental effect of HD and PD. Researchers need to explore the links between climate change and adverse health outcomes in patients with CKD. We need not wait for the results of studies to begin a concerted effort to reduce the carbon footprint of the dialysis industry. Medical directors of dialysis clinics must advocate to reduce waste, upgrade water systems, and implement clinic-specific practices to minimize energy consumption and adopt renewable energy. Physicians need to prepare patients for extreme weather and educate them about the risks of rising temperatures.
16	“Addressing the Environmental Impact of Kidney Care”	Yau A. et al. (2021) [11]	Australia	x	x	x	x	The efficiency of HD machines and reverse osmosis systems has improved somewhat, although they remain water- and power-intensive. Consumable use for both HD and PD also remains high, with most items designed for single use and without the ability to be recycled, be repurposed, or to biodegrade at the end of life.	Implement collaboration between the nephrology community, industry groups, and other disciplines with the aim of finding solutions.
17	“Green nephrology and eco-dialysis: a position statement by the Italian Society of Nephrology”	Piccoli G.B. et al. (2020) [24]	Italy and France		x	x	x	Green dialysis can be achieved by: 1. Reducing the burden of dialysis; 2. Favoring natural medicine; 4. Reuse and recycling of paper, glass and non-contaminated plastic; 6. Reducing water and energy consumption; 7. Introducing environmental impact criteria in checklists when evaluating dialysis machines and supplies; 8. Demanding planet-friendly approaches in the building of new facilities.	
